# Galectin-1 accelerates high-fat diet-induced obesity by activation of peroxisome proliferator-activated receptor gamma (PPARγ) in mice

**DOI:** 10.1038/s41419-020-03367-z

**Published:** 2021-01-11

**Authors:** Jung-Hwan Baek, Da-Hyun Kim, Jaegyeong Lee, Seok-Jun Kim, Kyung-Hee Chun

**Affiliations:** 1 Department of Biochemistry and Molecular Biology, Yonsei University College of Medicine, Seoul, South Korea; 2 Brain Korea 21 Project for Medical Science, Yonsei University, 50 Yonsei-ro, Seodaemun-gu, Seoul, 03722 Republic of Korea; 3 Department of Biomedical Laboratory Science, College of Medical Sciences, Daegu Hanny University, 1 Haanydae-ro, Gyeongsan-si, Gyeongsangbuk-do 38610 Republic of Korea; 4 Department of Biomedical Science & BK21 FOUR Educational Research Group for Age-associated Disorder Control Technology, Chosun University, Gwangju, 61452 Republic of Korea

**Keywords:** Glycoconjugates, Obesity

## Abstract

Galectin-1 contains a carbohydrate-recognition domain (CRD) as a member of the lectin family. Here, we investigated whether galectin-1 regulates adipogenesis and lipid accumulation. Galectin-1 mRNA is highly expressed in metabolic tissues such as the muscle and adipose tissues. Higher mRNA expression of galectin-1 was detected in white adipose tissues (WATs) of mice that were fed a high-fat diet (HFD) than in those of mice fed a normal-fat diet (NFD). Protein expression of galectin-1 also increased during adipocyte differentiation. Galectin-1 silencing inhibited the differentiation of 3T3-L1 cells and the expression of lipogenic factors, such as PPARγ, C/EBPα, FABP4, and FASN at both mRNA and protein levels. Lactose, an inhibitor by the binding with CRD of galectin-1 in extracellular matrix, did not affect adipocyte differentiation. Galectin-1 is localized in multiple cellular compartments in 3T3-L1 cells. However, we found that DMI (dexamethasone, methylisobutylxanthine, insulin) treatment increased its nuclear localization. Interestingly, galectin-1 interacted with PPARγ. Galectin-1 overexpression resulted in increased PPARγ expression and transcriptional activity. Furthermore, we prepared galectin-1-knockout (*Lgals1*^−*/*−^) mice and fed a 60% HFD. After 10 weeks, *Lgals1*^−/−^ mice exhibited lower body weight and gonadal WAT (gWAT) mass than wild-type mice. Fasting glucose level was also lower in *Lgals1*^−/−^mice than that in wild-type mice. Moreover, lipogenic genes were significantly downregulated in the gWATs and liver tissues from *Lgals1*^−/−^ mice. Pro-inflammatory cytokines, such as CCL2, CCL3, TNFα, and F4/80, as well as macrophage markers, were also drastically downregulated in the gWATs and liver tissues of *Lgals1*^−/−^ mice. In addition, *Lgals*1^−/−^mice showed elevated expression of genes involved in thermogenesis in the brown adipose tissue. Collectively, galectin-1 exacerbates obesity of mice fed HFD by increment of PPARγ expression and activation. Our findings suggest that galectin-1 could be a potential therapeutic target for obesity and needed further study for clinical application.

## Introduction

Galectins are a family of proteins that bind to β-galactoside. All galectins have a conserved carbohydrate-recognition domain (CRD), consisting of approximately 130 amino acids^1^. Galectins are localized in extra- and intra- cellular compartments, and are involved in cell adhesion, cell cycle progression, apoptosis, inflammation, and cell growth^2^. Most studies on galectins have focused on cancer and inflammatory disorders^3^. Galectins are also involved in metabolic diseases such as obesity and diabetes^4^. For example, the role of galectin-12^4,5^ and galectin-3^6^ in adipocyte differentiation and obesity has been already investigated.

Galectin-1 has been studied for its involvement in cancer progression and inflammation^7,8^. Galectin-1 overexpression promotes cell transformation by increasing membrane anchorage and oncogenic H-RAS signal transduction^9^. Galectin-1 induces apoptosis in activated CD4^+^ and CD8^+^ T cells^10^, and promotes angiogenesis by stimulating vascular endothelial cell proliferation and migration^11^. Interestingly, a previous study was investigated galectin-1 as a novel adipokine during adipocyte differentiation^12^. Galectin-1 is also known as a lipid droplet-associated protein in primary mouse adipocytes^13^. A recent report demonstrated that treating 3T3-L1 cells with thiodigalactoside (TDG), an inhibitor of galectins, retarded adipocyte differentiation. In addition, TDG-treated rats exhibited reduced body weight and white adipose tissue (WAT) mass, suggesting that TDG treatment increases the resistance to high-fat diet (HFD)-induced obesity^14^. These findings suggested that galectin-1 might be positively associated with adipocyte differentiation and obesity. However, the underlying molecular mechanisms are not yet fully understood.

In this study, we demonstrated that galectin-1 was highly expressed in the mouse adipose tissue and further increased during adipocyte differentiation and lipid accumulation. Therefore, we explored the mechanism by which galectin-1 regulates adipogenesis and obesity. To this end, preadipocyte 3T3-L1 cells were used, and the metabolic phenotype of galectin-1-deficient (*Lgals1*^−/−^) mice exposed to an obesogenic HFD was characterized.

## Results

### Galectin-1 is upregulated during adipocyte differentiation and predominantly expressed in mouse adipose tissue

**Fig. 1:**
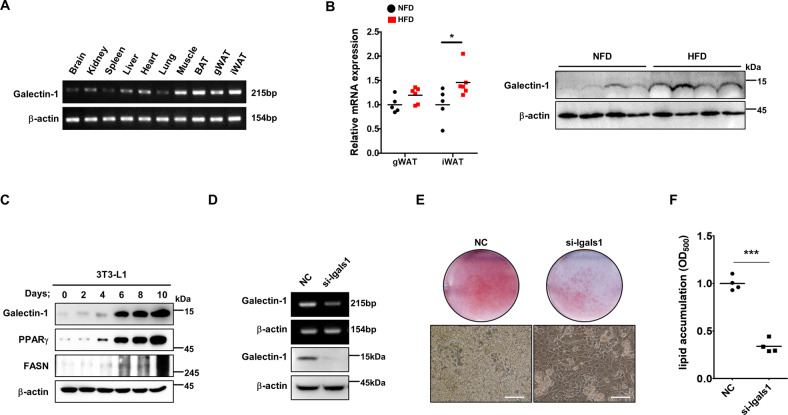
Galectin-1 expression during adipocyte differentiation and in mouse adipose tissues. **a** The expression of galectin-1 mRNA in mouse tissues was analyzed by RT-PCR. β-actin mRNA expression was used as a loading control. **b** The level of galectin-1 mRNA and protein was analyzed by quantitative RT-PCR and western blotting in gonadal white adipose tissues (gWATs) and inguinal (iWATs) from mice fed a normal-fat diet (NFD) or a high-fat diet (HFD) for 12 weeks. Data are presented as the mean ± standard error of the mean (SEM). *p < 0.05 for the NFD group (*n* = 5) vs. the HFD group (*n* = 6). **c** The protein level of galectin-1 and lipogenic proteins, such as PPARγ and FASN was analyzed by western blotting during DMI (dexamethasone, methylisobutylxanthine, insulin) induced differentiation of preadipocyte 3T3-L1 cells. β-actin expression was used as a loading control. **d** The expression level of galectin-1 mRNA and protein was analyzed by RT-PCR (two upper panels) and western blotting (two lower panels) after galectin-1 knockdown by a specific siRNA in 3T3-L1 cells. The mRNA and protein expression levels were normalized to those of β-actin. **e** Detection of adipocyte differentiation of 3T3-L1 cells following galectin-1 silencing. The cells were treated with galectin-1 siRNA and differentiation was induced by DMI treatment for 6 days. Oil red o (ORO) staining was performed to detect lipid accumulation (upper figures) and the staining level was taken by picture. Cell morphology was detected (lower figures) by microscopy. Bars indicated 50 μm. **f** Measurement of lipid accumulation. The ORO dye (used for staining) was eluted with 100% isopropanol and the optical density at 500 nm (OD_500_) was detected. ***p < 0.001 for the NC group vs. the galectin-1 siRNA group.

To determine the in vivo distribution of galectin-1, various mouse organs were examined by RT-PCR analysis (Fig. 1a). Galectin-1 was more highly expressed in metabolic tissues, such as the muscle and adipose tissue, than in other organs. Moreover, the level of galectin-1 mRNA and protein was higher in white adipose tissues from obese mice, which were fed a 60% high-fed diet (HFD) for 12 weeks, compared to those of normal-fat diet (NFD) fed mice (Fig. 1b).

We measured the changes in galectin-1 expression levels during the differentiation of pre-adipocyte 3T3-L1 cells to adipocytes (Fig. 1c). The cells were treated with DMI (dexamethasone, methylisobutylxanthine, insulin) and differentiated for 10 days. Galectin-1 expression, as well as the expression of peroxisome proliferator-activated receptor gamma (PPARγ) and fatty acid synthase (FASN), two markers of adipocyte differentiation, increased during adipocyte differentiation.

To study the role of galectin-1 in adipocyte differentiation, the corresponding gene was silenced by transfecting 3T3-L1 cells with a galectin-1-specific small interfering RNA (siRNA), followed by cell differentiation for 6 days (Fig. 1d). Galectin-1-depleted 3T3-L1 cells showed significantly delayed adipocyte differentiation and lipid accumulation, compared to control cells (Fig. 1e). Lipid accumulation was also significantly dropped by galecin-1 depletion at day 6 (Fig. 1f). These results suggested that galectin-1 regulated adipocyte differentiation and lipid accumulation.

### Galectin-1-depleted 3T3-L1 cells exhibit retarded adipocyte differentiation

**Fig. 2:**
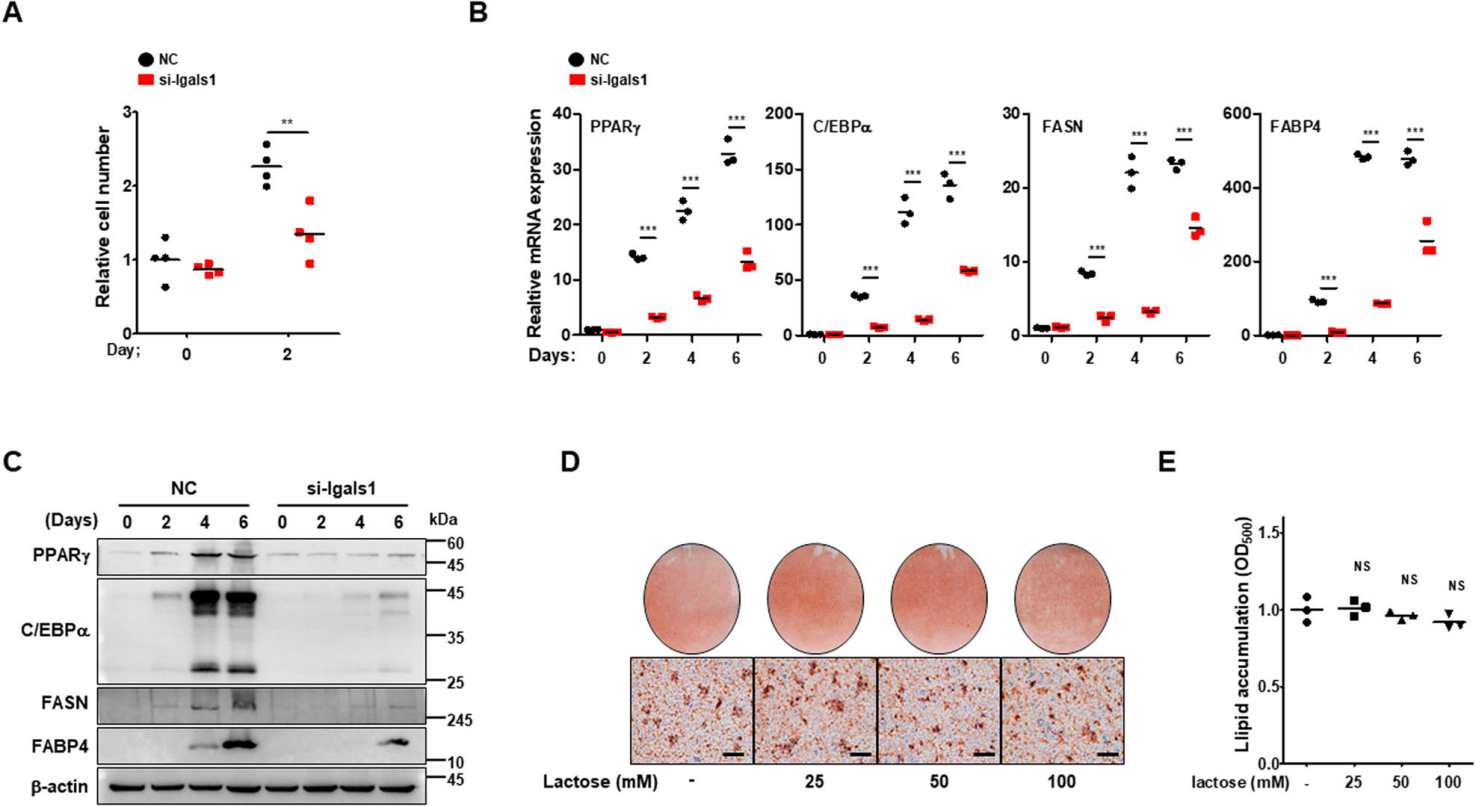
Galectin-1 knockdown inhibited adipocyte differentiation. **a** Detection of mitotic clonal expansion during adipocyte differentiation. Cells were counted by viewing with microscopy at 0 and 48 h after inducing adipocyte differentiation. **b** The mRNA expression level of adipogenic and lipogenic factors, such as PPARγ, C/EBPα, FASN and FABP4 was analyzed by quantitative RT-PCR in 3T3-L1 cells, with or without galectin-1 silencing. Data are presented as the mean ± SEM (*n* = 3 for each lane). ***p < 0.001 for the NC group vs. the Lgals1 siRNA treated group. **c** The protein expression level of adipogenic and lipogenic factors was analyzed by western blotting in 3T3-L1 cells, with or without galectin-1 silencing. β-actin expression was used as a loading control. **d** Detection of adipocyte differentiation of 3T3-L1 cells in the absence or presence of lactose (25, 50, or 100 mM). Adipocyte differentiation was induced by treating the cells with DMI and lactose for 6 days. Cell morphology was detected by microscopy. Bars indicated 50 μm. **e** Measurement of lipid accumulation. The ORO dye (used for staining) was eluted with 100% isopropanol and the OD_500_ was measured.

Galectin-1 depletion reduced mitotic clonal expansion, which was measured 48 h after adipogenic induction (Fig. 2a). The impact of galectin-1 depletion on mRNA (Fig. 2b) and protein expression (Fig. 2c) of lipogenic factors, such as PPARγ, CCAAT enhancer binding protein alpha (C/EBPα), FASN, and fatty acid-binding protein 4 (FABP4), was also examined after adipocyte differentiation. The expression of these lipogenic factors was attenuated by galectin-1 depletion, at both mRNA and protein levels. These data suggested that galectin-1 was essential for adipocyte differentiation.

Next, the effect of extracellular-localized galectin-1 on adipocyte differentiation was investigated (Fig. 2d and e). Treatment of 3T3-L1 cells with lactose, an inhibitor of galectin-1 by binding CRD, at concentrations up to 100 mM did not affect cell differentiation. It suggested that galectin-1-mediated regulation of adipocyte differentiation did not occur through an extracellular CRD binding mechanism.

### Galectin-1 interacts with PPARγ and increases its expression and transcriptional activation

**Fig. 3:**
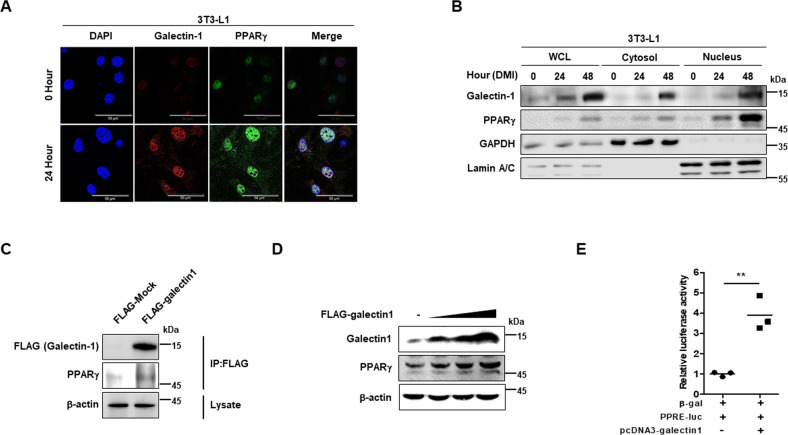
Galectin-1 interacted with PPARγ. **a** Detection of localization of galectin-1 and PPARγ after DMI treatment in preadipocyte 3T3-L1 cells by confocal microscopy. Galectin-1 was stained with cy5-conjugated antibody and PPARγ with FITC-conjugated antibody. DAPI was used for nuclear staining. Bars indicated 50 μm. **b** Cellular localization and expression level of galectin-1 and PPARγ were analyzed by western blotting using nuclear and cytosolic fractions of 3T3-L1 cells. GAPDH and Lamin A/C expression levels were detected and used as loading controls. **c** Immunoprecipitation of galectin-1 and PPARγ was performed in human embryonic kidney 293 (HEK293) cells. Cell lysates were immuneprecipitated using anti-Flag beads. β-actin expression was detected by western blotting as a loading control. **d** Flag-tagged galectin-1 overexpression vector was transfected in HEK293 cells and endogenous PPARγ expression was detected by western blotting. β-actin expression was used as a loading control. **e** Detection of luciferase activity of PPAR-response element (PPRE) by galectin-1 overexpression. Luciferase activity of PPRE was normalized to β-gal activity. Data are presented as the mean ± SEM. **p < 0.01 for the mock-transfected group vs. the galectin-1-overexpression group.

We examined the subcellular localization of galectin-1 during adipocyte differentiation (Fig. 3a and b). Total galectin-1 expression was found to increase after DMI treatment. Interestingly, the nuclear localization of galectin-1 significantly increased in a time-dependent manner, as did that of PPARγ (Fig. 3b). Co-localization of galectin-1 and PPARγ was detected in nucleus after DMI treatment (Fig. 3a).

Therefore, we performed immunoprecipitation assays and determined that galectin-1 and PPARγ interacted with each other (Fig. 3c). Overexpression of galectin-1 also increased the protein expression level of endogenous PPARγ (Fig. 3d). Moreover, overexpression of galectin-1 significantly increased the PPARγ transcriptional activation, indicated by the increment of PPAR-response element (PPRE) activation (Fig. 3e). These data suggested that DMI treatment increased galectin-1 expression in nucleus, and that galectin-1 interacted with PPARγ and promoted its expression and transcriptional activation.

### Lgals1^−/−^ mice show resistance to HFD-induced obesity

**Fig. 4:**
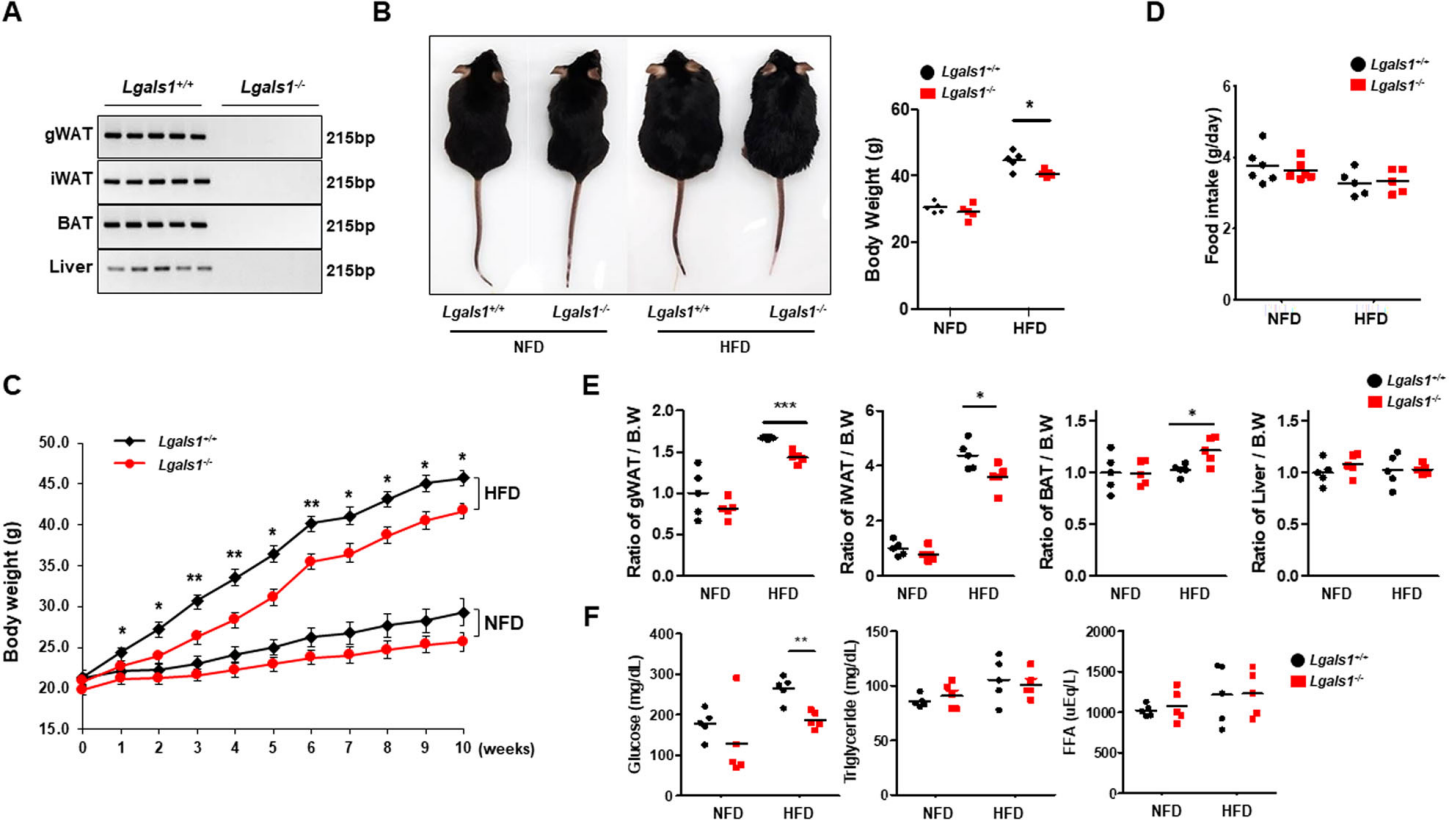
*Lgals1*^*−/−*^ mice were lean and showed reduced WAT masses. Both *Lgals1*^*+/+*^ and *Lgals1*^*−/−*^ mice were fed a normal-fat diet (NFD) or a high-fat diet (HFD) (60% fat) for 10 weeks (*n* = 5 per group). **a** Expression of galectin-1 mRNA was analyzed by RT-PCR in gonadal white adipose tissue (gWAT), inguinal white adipose tissue (iWAT), brown adipose tissue (BAT), and liver samples from a NFD fed *Lgals1*^*+/+*^ and *Lgals1*^*−/*−^ mice. **b** Body sizes and weights were measured and presented as a picture and a body weight graph. **c** The increment of body weight for 10 weeks was presented as a graph. **d** Daily food intake of *Lgals1*^*+/+*^ and *Lgals1*^*−/*−^ mice fed a NFD or a HFD. **e** Ratio of gWAT, iWAT, and BAT weights to body weights of *Lgals1*^*+/+*^ and *Lgals1*^−*/*−^ mice fed a NFD or a HFD. **f** Levels of serum glucose, triglycerides, and free fatty acids in *Lgals1*^*+/+*^ and *Lgals1*^*−/−*^ mice fed a NFD or a HFD. Data are presented as the mean ± SEM *p < 0.05, **p < 0.01, and ***p < 0.001 for the *Lgals1*^*+/+*^ mice vs. the *Lgals1*^*−/−*^ mice.

To confirm the role of galectin-1 in an in vivo mouse model of obesity, male wild-type (*Lgals1*^+/+^) and galectin-1 knockout (*Lgals1*^−/−^) mice (*n* = 5 per group) were fed a high-fat diet (HFD) containing 60% fat for 10 weeks (Fig. 4a). The body weights were not significantly different between those from *Lgals1*^−/−^ mice and wild-type mice fed normal-fat diet (NFD) (Fig. 4b and c). However, *Lgals1*^−/−^ mice exhibited smaller body size (Fig. 4b) and lower body weights than those of wild-type mice after exposure to the HFD (Fig. 4c), even though the food intake did not significantly differ between the two groups (Fig. 4d). The weight of mouse tissues involved in fat metabolism, i.e., WAT, brown adipose tissue (BAT), and liver tissues, was also measured (Fig. 4e). Both gWAT and iWAT as per body weight ratio were decreased in HFD-fed *Lgals1*^*−/*−^ mice, compared to those from HFD-fed wild-type mice. Interestingly, the relative BAT weight increased in *Lgals1*^−/−^ mice fed a HFD. No differences were observed in liver weight between the groups. Moreover, because obesity is known to increase the risk of type-2 diabetes mellitus (T2D) and hyperlipidemia, the serum levels of glucose, triglycerides, and free fatty acids were determined (Fig. 4f). The fasting glucose level was lower in HFD-fed *Lgals1*^−/−^ mice than HFD-fed wild-type mice, although the levels of triglycerides and free fatty acids did not differ.

### *Lgals1*^−/−^ mice exhibit decreased adiposity and altered expression of genes involved in lipid metabolism and thermogenesis

**Fig. 5:**
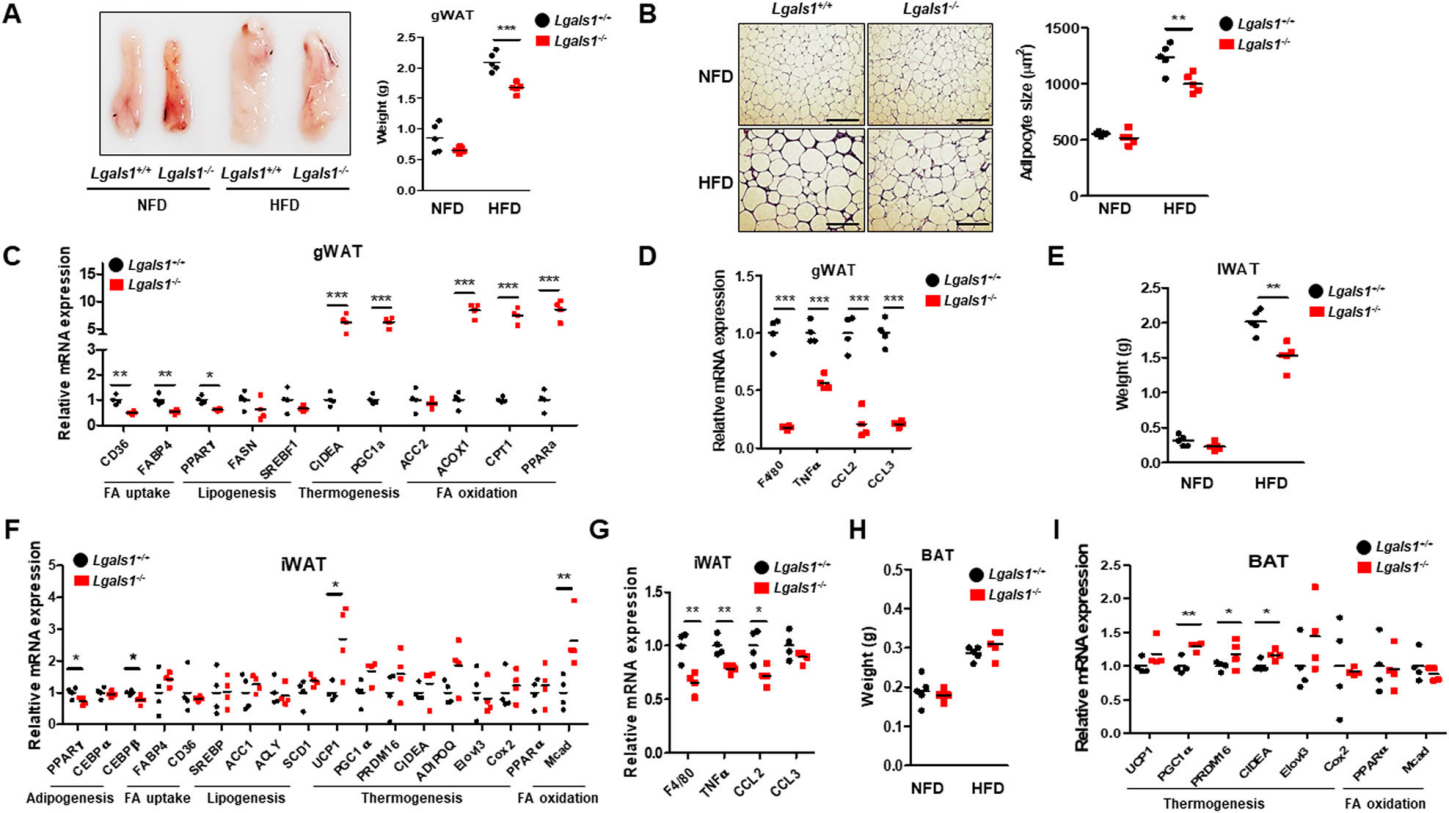
*Lgals1*^*−/−*^ mice had decreased expression of lipogenic genes and increased expression of thermogenic genes in adipose tissues. Both *Lgals1*^*+/+*^ and *Lgals1*^*−/−*^ mice were fed a NFD or a HFD for 10 weeks (*n* = 5 per group) and their gWATs (**a**–**d**), iWATs (**e**–**g**) and BATs (**h** and **i**) were prepared for experiments. **a** Size and weight of gWATs in *Lgals1*^*+/+*^ and *Lgals1*^*−/−*^ mice fed a NFD or a HFD were measured and presented as a picture and a graph. **b** Slide sections of gWAT from *Lgals1*^*+/+*^ and *Lgals1*^*−/−*^ mice fed a NFD or a HFD were prepared and stained with H&E. Morphology of adipocytes was presented as a picture. Bars indicated 100 μm. Size of adipocytes was measured using Image J software and presented as a graph. **c** The expression of genes involved in fatty acid uptake, such as CD36 and FABP4, in lipogenesis, such as PPARγ, FASN, SREBF1, in thermogenesis, such as CIDEA and PGC1α, and in fatty acid oxidation, such as ACOX1, CPT1 and PPARα in gWATs from *Lgals1*^*+/+*^ and *Lgals1*^*−*^^*/−*^ mice fed a HFD was analyzed by quantitative RT-PCR. **d** The expression of genes, included F4/80, a macrophage marker and pro-inflammatory cytokines, such as TNFα, CCL2 and CCL3 in gWATs from *Lgals1*^*+/+*^ and *Lgals1*^*−/−*^ mice fed a HFD was analyzed by quantitative RT-PCR. Data are presented as the mean ± SEM ***p < 0.001 for the *Lgals1*^*+/+*^ mice vs. the *Lgals1*^*−/−*^ mice. **e** Weight of iWATs in *Lgals1*^*+/+*^ and *Lgals1*^*−/−*^ mice fed a NFD or a HFD was measured and presented as a graph. **f** The expression of genes involved in adipogenesis, such as PPARγ, CEBPα and CEBPβ, in fatty acid uptake, such as CD36 and FABP4, in lipogenesis, such as SREBP, ACC1, ACLY and SCD1, in thermogenesis, such as UCP1, PGC1α, PRDM16, CIDEA, ADIPOQ, Elovl3 and COX2, and in fatty acid oxidation, such as PPARα and Mcad in iWATs from *Lgals1*^*+/+*^ and *Lgals1*^*−/−*^ mice fed a HFD was analyzed by quantitative RT-PCR. **g** The expression of genes, included F4/80, TNFα, CCL2 and CCL3 in iWATs from *Lgals1*^*+/+*^ and *Lgals1*^*−/−*^ mice fed a HFD was analyzed by quantitative RT-PCR. Data are presented as the mean ± SEM *p < 0.05 and **p < 0.01 for the *Lgals1*^*+/+*^ mice vs. the *Lgals1*^*−/−*^ mice. **h** Weight of BATs in *Lgals1*^*+/+*^ and *Lgals1*^*−/−*^ mice fed a NFD or a HFD were measured and presented as a graph. **i** The expression of genes involved in thermogenesis, such as UCP1, PGC1α, PRDM16, CIDEA, Elovl3 and COX2, and in fatty acid oxidation, such as PPARα and Mcad in iBATs from *Lgals1*^*+/+*^ and *Lgals1*^*−/−*^ mice fed a HFD was analyzed by quantitative RT-PCR. Data are presented as the mean ± SEM *p < 0.05 and **p < 0.01 for the *Lgals1*^*+/+*^ mice vs. the *Lgals1*^*−/−*^ mice.

The gWATs of HFD-fed *Lgals1*^−/−^ mice exhibited smaller size and weight than those of wild-type mice (Fig. 5a). We observed that adipocytes in gWAT were smaller in *Lgals1*^*−/−*^ mice than those in wild-type mice (Fig. 5b). HFD-fed *Lgals1*^−/−^ mice also showed lower iWAT weight than that of their wild-type counterparts (Fig. 5e). The expression levels of genes, which were involved in fatty acid uptake, and lipogenesis were found to be significantly decreased in both gWATs and iWATs from HFD-fed *Lgals1*^−/−^ mice (Fig. 5c and f). Obesity is known to cause macrophage infiltration and elevated levels of pro-inflammatory cytokines in WATs^15^. Therefore, the expression of F4/80, a macrophage marker, and pro-inflammatory cytokines, such as TNFα, CCL2 and CCL3 was measured (Fig. 5d and g). It revealed that they were significantly downregulated in both gWATs and iWATs from HFD-fed *Lgals1*^−/−^ mice.

Interestingly, no difference was observed in BAT weight between *Lgals1*^−/−^ mice and wild-type mice when both the groups were fed NFD and HFD (Fig. 5h). However, the BAT weight ratio per body weight was higher in HFD-fed *Lgals1*^−/−^ mice than that of HFD-fed wild-type mice (Fig. 4e). Thermogenesis, fatty acid oxidation, and lipid accumulation and synthesis were previously reported to regulate the fat mass^16^. Therefore, the expression levels of genes involved in thermogenesis and fatty acid oxidation were measured in gWAT, iWAT and BAT. Not only in BAT of HFD-fed *Lgals1*^−/−^ mice, these genes were significantly upregulated in both gWAT and iWAT (Fig. 5c, f, and i). It suggested that depletion of galectin-1 increased thermogenic gene expression to lead resistance to obesity.

### The expression of genes promoting hepatic gluconeogenesis and lipogenesis is decreased in *Lgals1*^−/−^ mice

**Fig. 6:**
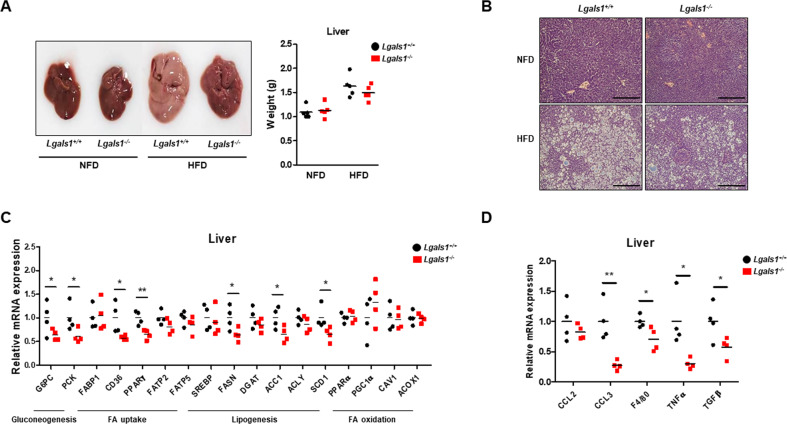
*Lgals1*^*−/−*^ mice showed the amelioration of fatty liver phenotype. Both *Lgals1*^*+/+*^ and *Lgals1*^*−/−*^ mice were fed a NFD or a HFD for 10 weeks (*n* = 5 per group). **a** Liver sizes and weights from *Lgals1*^*+/+*^ and *Lgals1*^*−/−*^ mice fed a NFD or a HFD were analyzed and presented as a picture and a graph. **b** Slide sections of liver from *Lgals1*^*+/+*^ and *Lgals1*^*−/−*^ mice fed a NFD or a HFD were prepared and stained with H&E. Bars indicated 100 μm. **c** The expression of genes involved in gluconeogenesis, such as G6PC and PCK, in fatty acid uptake, such as FABP1, CD36, PPARγ, FATP2 and FATP5, in lipogenesis, such as SREBP, FASN, DGAT, ACC1 ACLY and SCD1, and in fatty acid oxidation, such as PPARα PGC1α, CAV1 and ACOX1 in livers from *Lgals1*^*+/+*^ and *Lgals1*^*−/−*^ mice fed a HFD was analyzed by quantitative RT-PCR. **d** The expression of genes, included F4/80, CCL2, CCL3, TNFα and TGFβ in livers from *Lgals1*^*+/+*^ and *Lgals1*^*−/−*^ mice fed a HFD was analyzed by quantitative RT-PCR. Data are presented as the mean ± SEM *p < 0.05 and **p < 0.01 for the *Lgals1*^*+/+*^ mice vs. the *Lgals1*^*−/−*^ mice.

Because fatty liver diseases are often exhibited in obesity, we conducted histological analysis of the liver. No phenotypic differences were found in livers from both NFD-fed *Lgals1*^*−*^^/−^ mice and wild-type mice (Fig. 6a and b). However, while a remarkable fat accumulation was observed in the liver of HFD-fed wild-type mice, this phenomenon was substantially attenuated in HFD-fed *Lgals1*^*−/−*^ mice. However, differences of liver weights were detected slightly in the between *Lgals1*^−^/^−^ mice and wild-type mice, after HFD exposure (Fig. 6a). The expression of genes involved in gluconeogenesis, such as glucose-6-phosphatase (G6PC) and phosphoenolpyruvate carboxykinase (PCK), were significantly reduced in the livers of HFD-fed *Lgals1*^*−/−*^ mice (Fig. 6c). The expression of *FASN*, *ACC1*, and *SCD1* (genes involved in lipogenesis) were also downregulated in HFD-fed *Lgals1*^−*/*−^ mice (Fig. 6c). Moreover, macrophage markers (F4/80) and pro-inflammatory cytokines (TGFβ, TNFα, CCL2 and CCL3) were found to be significantly downregulated in the livers of HFD-fed *Lgals1*^*−*^^/−^ mice compared to their *Lgals1*^+/+^ counterparts (Fig. 6d). It suggested that depletion of galectin-1 significantly reduced fat accumulation and inflammatory cytokine secretion in liver.

## Discussion

The prevalence of obesity is rapidly growing worldwide. Obesity is known to increase the rate of mortality through an elevated risk of developing several chronic conditions, such as T2D, cardiovascular disease, osteoarthritis, dementia, and cancer, as well as to exacerbate the severity of various acute diseases^17^. Here, we addressed the role of galectin-1 in obesity. Galectins are involved in various aspects of biological homeostasis and their role in cancer and inflammatory diseases has been extensively investigated^18–21^. Galectins also represent a potential therapeutic target for obesity^17^. The role of galectin-1 in obesity has also been also explored^14,22^. However, the exact function of galectin-1 in the context of obesity is still unknown.

In this study, we observed that higher expression of galectin-1 was detected in adipose tissues of HFD-fed mice than in those of NFD-fed mice. Galectin-1 also increased during adipocyte differentiation and lipid accumulation^12^. It was reported that treatment with thiogalactoside (TDG)^14^ or lactulose^22^ reduced adipocyte differentiation and obesity in murine fed a HFD. TDG and lactulose is a non-metabolized disaccharide that can bind to galectins, and inhibit the function of intracellular and extracellular galectins^23^. In this study, the inhibition of extracellular galectins by lactose did not affect adipocyte differentiation. It was reported that extracellular galectin-1 stimulates angiogenesis in vitro and in vivo, and these effects were inhibited by lactose treatment^11^. Our finding suggested that the regulation of adipocyte differentiation by galectin-1 was based on an intracellular mechanism. Next, we determined that the expression of galectin-1 protein increased in both the nucleus and cytoplasm after induction of adipocyte differentiation. The increased nuclear localization of galectin-1 during adipocyte differentiation further supports this conclusion. Moreover, we provided the first evidence of an interaction between galectin-1 and PPARγ. Galectin-1 overexpression elevated PPARγ expression and PPRE activity. Based on these results, we suggested that nuclear galectin-1 may promote adipocyte differentiation by interacting with PPARγ, which is a major transcription factor modulating several biological processes that are perturbed in obesity, including inflammation, lipid and glucose metabolism, and overall energy homeostasis^24^. The high relevance of these processes explains the strong interest in PPAR agonists and antagonists in medical research and drug discovery^25^. Thus, our data identify galectin-1 as a potential regulator of PPARγ. It is also suggested that targeting galectin-1 may represent a new way to treat a wide spectrum of metabolic diseases by modulating PPARγ activity.

We further determined the metabolic phenotype in galectin-1-deficient (*Lgals1*^−/−^) mice that were fed a 60% high-fat diet (HFD). *Lgals1*^*−*^^/−^ mice showed decreased body weights and adipose tissues mass compared to those of wild-type (*Lgals1*^*+/+*^*)* mice, following HFD administration. Downregulation of genes involved in adipogenesis, fatty acid uptake, and lipogenesis were detected in both gWATs and iWATs of *Lgals1*^*−/−*^ mice. Interestingly, the expression of genes involved in thermogenesis and fatty acid oxidation were increased in WATs and BATs from *Lgals1*^*−/−*^ mice. Obesity causes decreased thermogenesis and fatty acid oxidation^26,27^. Therefore, these two processes are potential therapeutic targets for obesity^28–30^. In *Lgals1*^*−/−*^ mice, decreased lipogenesis, as well as increased thermogenesis and fatty acid oxidation, resulted in a synergistic inhibition of body weight gain. Further studies should be conducted to establish whether the increase in thermogenesis induced by galectin-1 knockdown involves the β-adrenergic signaling, which typically regulates the expression of thermogenic genes^28,29^.

*Lgals1*^−/−^ mice exhibited lower fasting glucose levels than those in the wild-type counterparts, supposed to be resulted in an improvement of diabetes. The expression of macrophage markers and pro-inflammatory cytokines were also decreased in the adipose tissues of *Lgals1*^*−/−*^ mice. Insulin resistance in obesity is known to be associated with an increased inflammation in adipose tissue^31,32^. The reduced inflammation observed in the adipose tissues of *Lgals1*^*−*^^*/−*^ mice suggested that galectin-1 targeting improved insulin resistance. A previous study conducted in non-obese diabetic mice showed that treatment with soluble galectin-1 promotes the apoptosis of pathological Th1 cells, causing pancreatic β-cell destruction^33^. Galectin-1 deficiency results in pro-diabetic effects, suppressing the function of pancreatic B-cells and increasing inflammation of the adipose tissues. In this study, we focused on lipogenesis and lipid accumulation in the adipose tissue, but did not explore the function of pancreatic β-cells or insulin resistance. Further studies focusing on the role of galectin-1 in glucose homeostasis will be necessary to clarify the impact of galectin-1 on diabetes.

We also found that *Lgals1*^*−/−*^ mice liver have lower expression of genes involved in gluconeogenesis, lipogenesis, and inflammation. Furthermore, we found that hepatic triglyceride accumulation was also reduced in *Lgals1*^−*/*−^ mouse livers. It is unclear whether this result reflects direct regulation by galectin-1 or an additional effect of obesity resistance. A recent report showed that the interaction of galectin-1 with neuropilin-1 promotes liver fibrosis by activating the hepatic stellate cells^34^. In addition, galectin-1 silencing was shown to inhibit the activation and proliferation of mouse hepatic stellate cells in a mouse model of liver fibrosis^35^. These results suggest that galectin-1 may have a direct impact on both obesity and liver disease.

Taken together, our findings suggest that galectin-1 expression increases during adipogenesis and accelerates adipocyte differentiation and lipid accumulation. We demonstrated that galectin-1 interacts with PPARγ and promoted its expression and transcriptional activity. Deletion of galectin-1 attenuated the effects of HFD on blood glucose levels and inflammation and reduced the body mass. In light of our results, the development of galectin-1 inhibitors should be considered as a therapeutic strategy for obesity and other metabolic diseases. Further studies are needed on this subject for their development.

## Materials and methods

### Cell culture and adipocyte differentiation

3T3-L1 and human embryonic kidney 293 (HEK293) cells were purchased from the Korea Cell Line Bank (Seoul, Korea). 3T3-L1 cells were maintained and differentiated as previously described^36,37^. Briefly, the cells were maintained in Dulbecco’s modified Eagle’s medium (DMEM; Welgene, Korea) supplemented with 10% calf serum and 1% antibiotics at 37 °C in 5% CO_2_. Confluent 3T3-L1 cells were incubated for 48 h. Then, the medium was replaced with DMEM supplemented with 10% fetal bovine serum (FBS), dexamethasone (1 µM), insulin (1 µg/ml), and isobutylmethylxanthine (520 µM). After 48 h, the medium was replaced with DMEM supplemented with 10% FBS and insulin (1 µg/ml), and after an additional 48 h, the medium was replaced with DMEM supplemented with 10% FBS. HEK293 cells were maintained in DMEM supplemented with 10% FBS and 1% antibiotics at 37 °C in 5% CO_2._

### RNA isolation and quantitative RT-PCR

Total RNA was prepared with the RNA lysis reagent (Intron Biotechnology, Korea), following the manufacturer’s instructions. Complementary DNA (1 µg) was synthesized using quantitative PCR master mix (TOYOBO, Osaka, Japan). The following primer sets were used for quantitative RT-PCR: ACC1, forward: 5′-ATGCGATCTATCCGTCGGTG-3′ and reverse: 5′-TCCTCCAGGCACTGGAACAT-3′; ACLY, forward: 5′-GAAGCTGACCTTGCTGAACC-3′ and reverse: 5′-CTGCCTCCAATGATGAGGAT-3′; adiponectin, forward: 5′-TACTGCAACATTCCGGGACTC-3′ and reverse: 5′-GAGGCCTGGTCCACATTCTT-3′; β-Actin, forward: 5′-GGCTGTATTCCCCTCCATCG-3′ and reverse: 5′-CCAGTTGGTAACAATGCCATGT-3′; C/EBPα, forward: 5′-GACATCAGCGCCTACATCGA-3′ and reverse: 5′-TCGGCTGTGCTGGAAGAG-3′; CCL2, forward: 5′-TAAAAAACCTGGATCGGAACCAA-3′ and reverse: 5′-GCATTAGCTTCAGATTTACGGGT-3′; CCL3, forward: 5′-GTGACTCACCTTGTGGTCCT-3′ and reverse: 5′-AGGGCAGATCCCAATTGTCAG-3′; CD36, forward: 5′-TGATACTATGCCCGCCTCTCC-3′ and reverse: 5′-TTTCCCACACTCCTTTCTCCTCTA-3′; CIDEA, forward: 5′-CATACATCCAGCTCGCCCTT-3′ and reverse: 5′-CGTAACCAGGCCAGTTGTGA-3′; F4/80, forward: 5′-CGTCAGCCGATTTGCTATCT-3′ and reverse: 5′-CGGACTCCGCAAAGTCTAAG-3′; FABP4, forward: 5′-CATCAGCGTAAATGGGGATt-3′ and reverse: 5′-TCGACTTTCCATCCCACTTC-3′; FASN, forward: 5′-TGGGTTCTAGCCAGCAGAGT-3′ and reverse: 5′-ACCACCAGAGACCGTTATGC-3′; G6PC, forward: 5′-CCTGAGGTACCAAGGGAGGA-3′ and reverse: 5′-GAAGGCGTTCCTCAGGTCAG-3′; galectin-1, forward: 5′-CTCTCGGGTGGAGTCTTCTG-3′ and reverse: 5′-GCGAGGATTGAAGTGTAGGC-3′; PCK, forward: 5′-AGATCATCATGCACGACCCC-3′ and reverse: 5′-TGTCCTTCCGGAACCAGTTG-3′; PGC1α, forward: 5′-ATGTGTCGCCTTCTTGCTCT-3′ and reverse: 5′-ATCTACTGCCTGGGGACCTT-3′; PPARγ, forward: 5′-AGGGCGATCTTGACAGGAAA-3′ and reverse: 5′-CGAAACTGGCACCCTTGAAA-3′; PRDM16, forward: 5′-CAGCACGGTGAAGCCATTC-3′ and reverse: 5′-GCGTGCATCCGCTTGTG-3′; SCD1, forward: 5′-GTACCGCTGGCACATCAACT-3′ and reverse: 5′-AAGCCCAAAGCTCAGCTACTC-3′; SREBP, forward: 5′-GATCAAAGAGGAGCCAGTGC-3′ and reverse: 5′-TAGATGGTGGCTGCTGAGTG-3′; TGFβ, forward: 5′-CCTGCAAGACCATCGACATG-3′ and reverse: 5′-TGTTGTACAAAGCGAGCACC-3′; TNFα, forward: 5′-CGTCAGCCGATTTGCTATCT-3′ and reverse: 5′-CGGACTCCGCAAAGTCTAAG-3′; and UCP1, forward: 5′-GGGCCCTTGTAAACAACAAA-3′ and reverse: 5′-GTCGGTCCTTCCTTGGTGTA-3′. Quantitative RT-PCR was performed using SYBR Premix Ex Taq (Clontech Laboratories, Mountain View, CA, USA) with ABI instruments (Applied Biosystems, Inc., Foster City, CA, USA). All expression results were normalized to β-Actin expression.

### Oil Red O (ORO) staining

Differentiated 3T3-L1 cells were washed with Dulbecco’s phosphate-buffered saline (PBS) and incubated in 10% formalin for 10 min. Next, the cells were washed with distilled water, and then with 60% isopropanol, and completely dried. The ORO stock solution (0.35 g/100 ml) was diluted with isopropanol to prepare a 60% ORO working solution. The dried cells were stained with the ORO working solution for 30 min and washed three time with distilled water.

### siRNA transfection

3T3-L1 cells were transfected with mouse galectin-1 siRNA (50 nM) using Lipofectamine RNAiMAX (Invitrogen, Carlsbad, CA, USA), according to the manufacturer’s protocol. After 24 h, the medium was replaced with maintenance medium supplemented with 10% calf serum^38,39^.

### Western blot analysis

Cell lysates were prepared by using RIPA buffer (Biosesang, Korea) containing a protease inhibitors cocktail (GeneDEPOT, Barker, TX, USA) and phosphatase inhibitor, incubated for 20 min on ice and centrifuged at 4 °C for 25 min at 13 200 revolutions per minute (rpm). Each supernatant was transferred to a new microcentrifuge tube. The protein concentration in each supernatant was measured with the protein assay reagent (Thermo Scientific, Waltham, MA, USA). Protein samples were resolved by sodium dodecyl sulfate (SDS)-polyacrylamide gel electrophoresis and transferred to 0.45-µm polyvinylidene fluoride membranes (Merck Millipore, Billerica, MA, USA). The membranes were blocked with 5% skim milk for 1 h at room temperature. After blocking, the membranes were incubated overnight at 4 °C with primary antibodies against galectin-1, PPARγ, C/EBPα, FASN, FABP4, GAPDH, Lamin A/C or β-Actin (Santa Cruz Biotechnology, Dallas, TX, USA) or against the Flag epitope (Sigma-Aldrich, St. Louis, Missouri, USA). The membranes were washed thrice for 10 min with PBS containing Tween 20 (PBST), and incubated for 1 h at room temperature with the appropriate horseradish peroxidase-conjugated secondary antibodies (Bethyl Laboratories, Montgomery, TX, USA). Next, the membranes were washed thrice for 10 min with PBST. The FUSION SOLO S Imaging System (Vilber, Eberhardzell, Germany) was used for detection, according to manufacturer’s directions. β-Actin was used as a loading control.

### Fractionation of cellular extracts

Nuclear and cytoplasmic extracts were prepared as previously described^40^. 3T3-L1 cells were lysed in Buffer A (10 mM HEPES (pH 7.9), 1.5 mM MgCl_2_, 10 mM KCl, 1 mM DTT, 0.2 mM phenylmethylsulfonyl fluoride (PMSF), 0.1% NP-40), incubated for 15 min on ice and centrifuged at 4 °C for 10 min at 850 relative centrifugal force (rcf). Each supernatant (Cytosol) was transferred to a new microcentrifuge tube. Remained pellets were lysed in Buffer C (20 mM HEPES (pH 7.9), 25% glycerol, 0.42 M NaCl, 0.2 mM EDTA, 1.5 mM MgCl_2_, 1 mM DTT, 0.2 mM PMSF) and vortexed for 15 sec. The lysates were incubated for 30 min on ice and vortexed every 10 min for 15 sec. After centrifugation (4 °C for 10 min at 13 200 rpm), Each supernatant (Nucleus) was transferred to a new microcentrifuge tube. Nuclear and cytoplasmic extracts were analyzed by western blotting. GAPDH and Lamin A/C expression levels were detected and used as loading controls.

### Immunoprecipitation

Cell lysate extraction was performed with immunoprecipitation buffer as previously^6^. After centrifugation (4 °C for 25 min at 13 200 rpm), the supernatants were mixed with protein A/G agarose beads (Santa Cruz Biotechnology), and precleared by incubation at 4 °C for 30 min in a rotor. After centrifugation (4 °C for 25 min at 13 200 rpm), anti-Flag beads (Sigma-Aldrich) were added to the supernatants, followed by an overnight incubation at 4 °C in a rotor. The immunoprecipitates were washed twice in immunoprecipitation buffer, mixed with 2× SDS sample buffer, and boiled at 100 °C for 5 min. After centrifugation (4 °C for 2 min at 13 200 rpm), the supernatants were analyzed by western blotting.

### Luciferase reporter assay

PPRE-TK-Luc, pcDNA3 galectin-1, and β-gal were co-transfected into HEK293 cells using Lipofectamine 2000 (Invitrogen). After 48 h, the cells were harvested and luciferase activity was measured using the Luciferase Assay System (Promega, Madison, Wisconsin, USA), according to the manufacturer’s directions. Luciferase activity was normalized using the β-gal Enzyme Assay System (Promega).

### Immunocytochemistry

3T3-L1 cells in chamber slides were fixed for 30 min with 10% formalin at 4 °C, washed with 1× PBS, and permeabilized in 0.5% Triton X-100 for 10 min. Next, the cells were incubated with primary antibodies at 4 °C, and then with fluorescein isothiocyanate-conjugated anti-mouse and cyanine 5-conjugated anti-rabbit secondary antibodies (Invitrogen), as well as 4′,6-diamidino-2-phenylindole (DAPI) staining solution (Vector Laboratories, Burlingame, CA, USA). The images were analyzed by confocal microscopy (LSM 700, Oberkochen, Germany).

### Mouse studies

The animal studies were approved by the Yonsei University Health System Institutional Animal Care and Use Committee (Permission number for animal experiments: 2015-0104). Galectin-1 knockout (*Lgals1*^−/−^ C57BL/6 mice were purchased from the Knockout Mouse Project Repository (Oakland, CA, USA). Wild-type (*Lgals1*^+/+^) and galectin-1 knockout (*Lgals1*^−/−^) mice were bred as heterozygotes in house and maintained on a C57BL/6 background. Heterozygous mice were bred with each other to generate *Lgals1*^+/+^ and *Lgals1*^−/−^ mice. Seven-week-old male *Lgals1*^+/+^ and *Lgals1*^*−*^^/−^ mice (5 per cage) were fed a NFD or a HFD (Research Diets, Inc., New Brunswick, NJ, USA) for 10 weeks. Littermate-matched mice were randomly divided into 4 groups. All mice were provided free access to food and water and kept on a 12 h light/12 h dark cycle. Body weight and food intake were measured once a week. After 10 weeks, mice were euthanized with CO_2_ gas. Mouse tissues and serum were stored at -80 °C before analysis. Portions of liver and WAT were fixed in 10% formalin for histological analysis.

### Immunohistochemical analysis of WATs and liver tissues

Mouse WATs and livers were fixed in 10% formalin and embedded in paraffin. Paraffin-embedded WAT or liver sections were subjected to hematoxylin and eosin (H&E) staining. H&E-stained sections were analyzed using Image J software^41^ (NIH, Bethesda, Maryland, USA).

### Statistical analysis

The unpaired (two-sample) *t*-test was used to determine the *p*-values. *P*-values < 0.05 were considered to reflect statistically significant differences. Statistical analysis was performed using Prism 5 (GraphPad software, La Jolla, CA, USA).
